# Limited Granulomatosis With Polyangiitis Presenting as Recurrent Lung Abscess Without Pneumonia

**DOI:** 10.7759/cureus.29858

**Published:** 2022-10-03

**Authors:** Venu Chippa, Rahul Gujarathi, Swetha Chenna, Narsimha Candula

**Affiliations:** 1 Internal Medicine, St. Vincent Medical Center, Evansville, USA; 2 Hospital Medicine, University of Florida Health, Jacksonville, USA; 3 Internal Medicine, Indiana University, Indianapolis, USA

**Keywords:** immunosuppression, limited gpa, pr3-anca, empyema, granulomatosis with polyangiitis

## Abstract

Granulomatosis with polyangiitis (GPA), previously called Wegener's granulomatosis, is a systemic necrotizing vasculitis affecting small and medium-sized vessels and is associated with antineutrophil cytoplasmic antibodies (ANCA). GPA is a systemic disease but can present in a limited form, where the respiratory system is the most commonly involved. Here, we report a case of a 54-year-old African American female who presented with chronic cough and got diagnosed with recurrent right-sided empyema without pneumonia. She underwent a right thoracotomy, and the biopsy showed necrotizing granulomatous inflammation with vasculitis and rare non-necrotizing granuloma, suggesting GPA. Diagnosis of GPA was confirmed by strongly positive anti-proteinase 3 ANCA antibodies. Interestingly, her GPA is a limited form, and she had an excellent recovery after initiation of immunosuppression. Early diagnosis and treatment are critical for better outcomes and survival in GPA.

## Introduction

Granulomatosis with polyangiitis (GPA) is a small and medium vessel necrotizing vasculitis associated with antineutrophil cytoplasmic antibody (ANCA). ANCA-associated vasculitis (AAV) includes granulomatosis with polyangiitis (GPA), microscopic polyangiitis (MPA), and eosinophilic granulomatosis with polyangiitis (EGPA or Churg-Strauss syndrome). GPA is potentially lethal, with a mortality rate of 20% if untreated [1].

GPA is a triad of the necrotizing upper and lower respiratory tract granulomas, systemic vasculitis, and necrotizing glomerulonephritis. However, in the limited form, it can present without systemic symptoms, the lung is the most commonly involved, and kidneys are almost always spared [2].

Among the three types of AAV, GPA is the most common. The annual worldwide incidence of GPA is estimated to be 10-20 cases per one million based upon geographical location [3]. The colder regions of the world have a higher incidence. The incidence in the United States is three cases per one million population. It has a peak incidence at 64-75 years of age, and recent studies have shown no sex predilection. It is commonly reported in Caucasians but is seen in all racial and ethnic groups [4].

We report an atypical presentation of a limited GPA in a middle-aged African American woman who presented with chronic dry cough and imaging showing recurrent empyema with a lung mass, eventually diagnosed with a limited GPA. This case emphasizes the importance of diagnosing a limited GPA with an atypical presentation. Timely diagnosis and early initiation of immunosuppression will improve the outcomes.

## Case presentation

**Figure 1:**
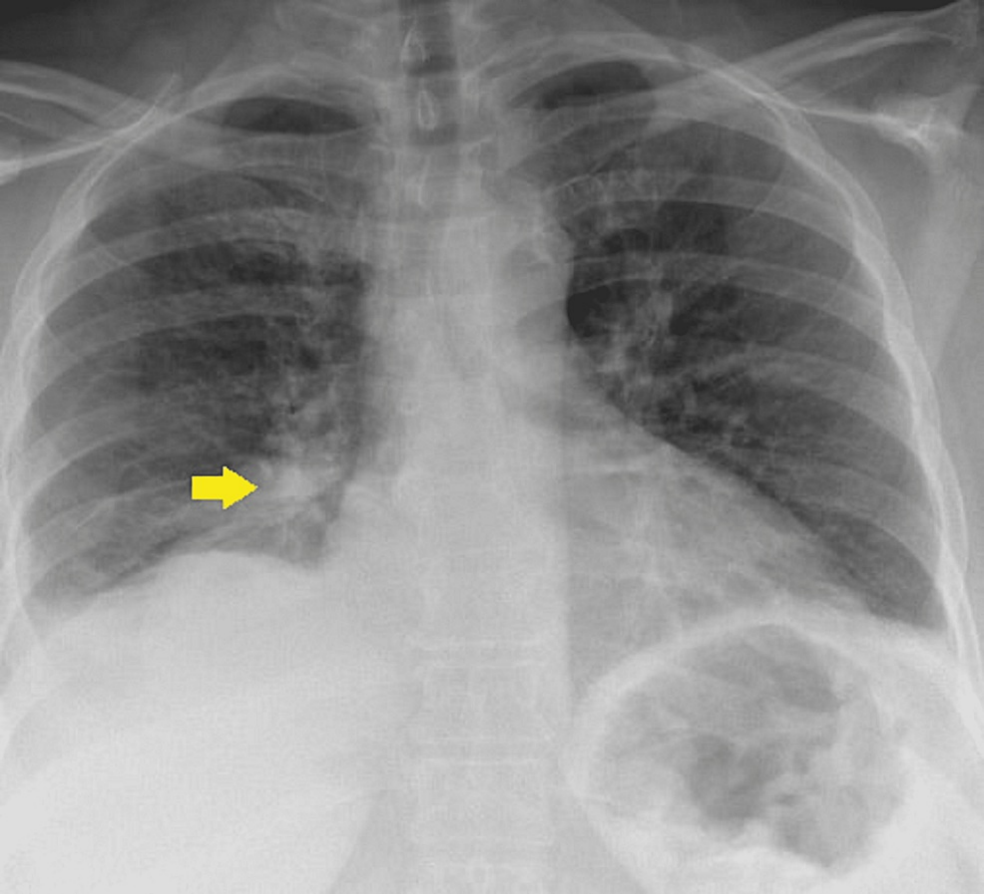
Chest x-ray showing right lung mass (arrow).

**Figure 2:**
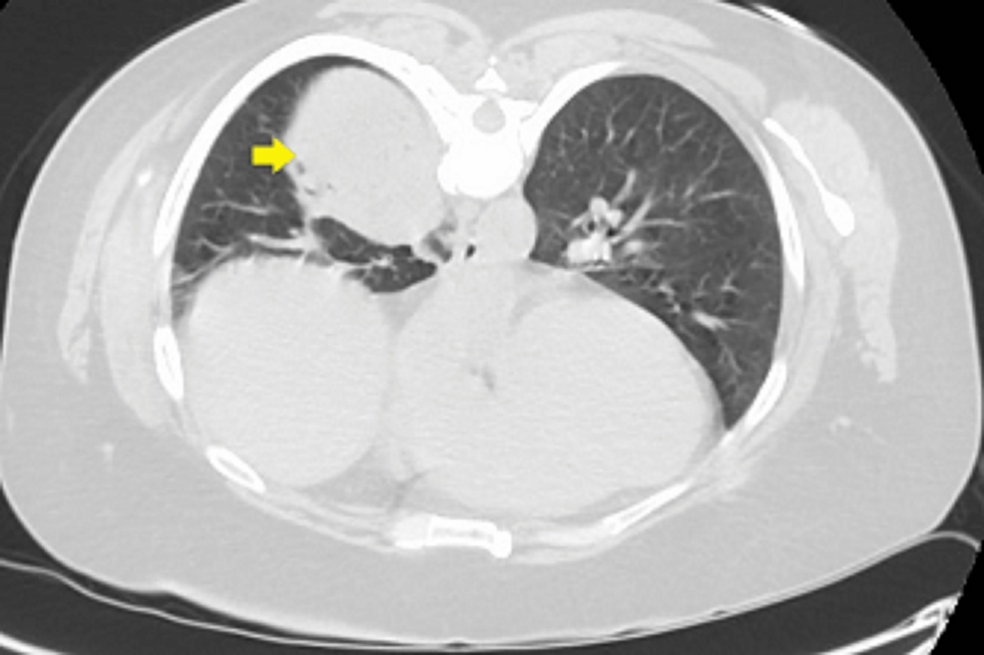
CT of the chest showing 5 x 3 x 5 cm lung mass abutting pleura (arrow).

A 54-year-old African American female was sent to the emergency room from the pulmonologist's office for right-sided loculated pleural effusion. She initially presented to the pulmonologist's office with complaints of shortness of breath and dry cough about four months ago. A chest x-ray showed a right lower lobe mass abutting the pleura (Figure 1). She got admitted to the hospital and underwent computerized tomographic (CT)-guided biopsy of the right lung mass (Figure 2).

**Figure 3:**
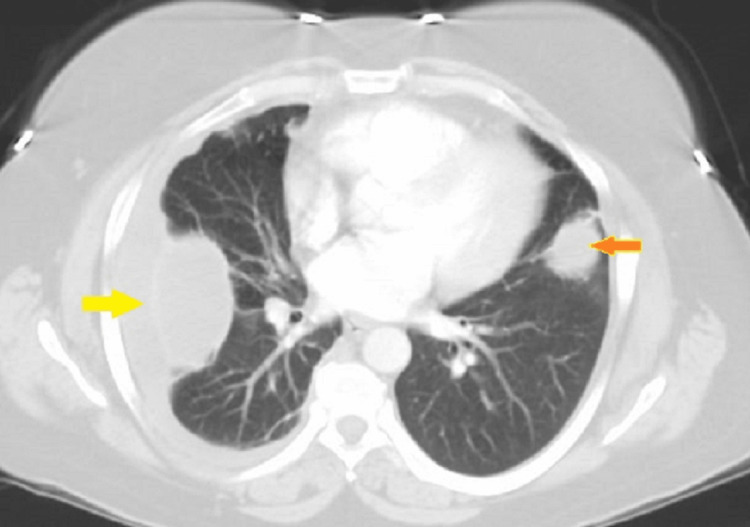
CT of the chest showing empyema and lung mass in the right lung (yellow arrow) and left lung nodule (orange arrow with yellow border).

The biopsy showed an abscess and was negative for malignancy. Microscopic examination was negative for bacteria and fungi and had negative pleural fluid and blood cultures. She received IV antibiotics in the hospital for two weeks and was discharged on oral clindamycin for another two weeks. One week after discharge, she returned to the hospital for worsening shortness of breath. A repeat chest CT scan showed new right-sided pleural effusion and development of nodular opacities in both lungs (Figure 3).

She was readmitted and underwent ultrasound-guided right-sided thoracentesis and required a chest tube; pleural fluid analysis was again consistent with empyema. She was again started on broad-spectrum intravenous antibiotics. The pleural fluid microscopy did not show any bacteria or fungi; all the cultures were negative.

Interestingly, her adenosine deaminase levels were elevated in the pleural fluid, but she had a negative QuantiFERON and tuberculin skin test. No acid-fast bacilli were seen on three sputum examinations. Finally, the patient was discharged on oral amoxicillin and clavulanic acid for two more weeks. At one-month follow-up, CT chest showed significant improvement in the lung nodules and a decrease in the size of the right lung mass.

**Figure 4:**
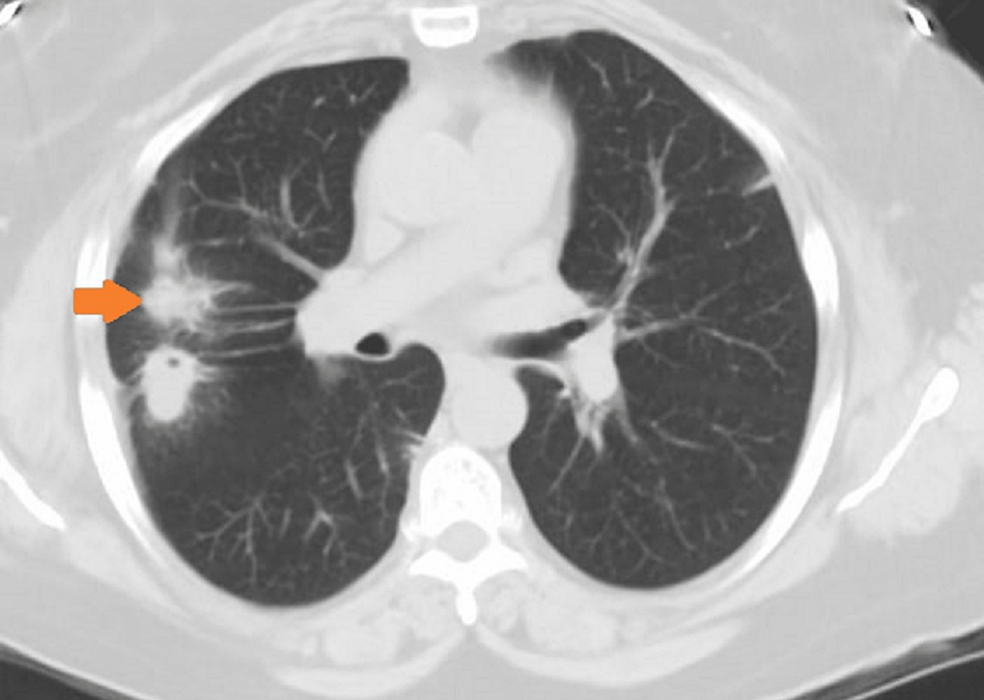
CT of the chest showing persistent right lung mass with possible abscess (arrow) and resolved left lung mass.

She returned to our office after a month as she was not feeling well. She continued to have some non-productive cough and fatigue, so a CT scan of the chest was ordered, and it showed an increase in the size of the right lower lobe mass compared to the previous CT scan of the chest (Figure 4).

Because of recurrent hospital admissions and negative pleural fluid analysis for infections and malignancy, she was sent to the hospital for admission and evaluation for video-assisted thoracoscopic surgery (VATS), decortication, and biopsy by cardiothoracic surgery.

She denied a history of fever, joint pains, runny nose, or oral ulcers in the emergency room. She had no known history of asthma, never-smoked, and had no exposure to tuberculosis. She was in no distress in the emergency room, and her vitals were stable. A systemic examination was normal except for absent air entry on the right lung base and dullness to percussion. Her complete blood count, basic metabolic panel, liver function test, and urinalysis were normal.

She was started on IV antibiotics, was seen by cardiothoracic surgery, and planned for VATS, biopsy, and lysis of adhesions. She had negative urine Histoplasma, Blastomyces, and serum cryptococcal antigen. She also had negative antinuclear antibodies, rheumatoid factor, and HIV serology. Again the cultures were negative, and histopathology examination showed necrotizing granulomatous inflammation with vasculitis.

Indirect immunofluorescence on ethanol-fixed neutrophils showed strong positivity for diffuse cytoplasmic staining of antineutrophil cytoplasmic antibody (C-ANCA) directed against proteinase 3 (PR 3) and negative anti-myeloperoxidase antibodies (MPO). Her c-reactive protein was within normal range. An erythrocyte sedimentation rate was slightly elevated in the range of 21-37 mm/h. She has been diagnosed with GPA affecting the lungs without the involvement of other organ systems. At discharge, she was advised to follow-up with the rheumatology office, where she was started on oral methotrexate and prednisone for induction. After a month, prednisone was discontinued, and she continued to be on methotrexate and folic acid for the next 10 months.

## Discussion

Granulomatosis with polyangiitis (GPA), formerly called Wegener's granulomatosis, is a rare systemic autoimmune necrotizing vasculitis affecting small and medium-sized vessels associated with antinuclear cytoplasmic antibodies (ANCA). GPA is one of the antineutrophil cytoplasmic antibodies (ANCA) associated with vasculitis (AAV) disorders. GPA has broad clinical manifestations from mild-to-severe disease. Though it initially presents as localized upper respiratory tract granulomatous disease, it can rapidly progress into more generalized vasculitis without proper treatment. Almost all patients have some involvement in the upper respiratory tract, 90% of patients have pulmonary involvement, and 80% of patients have renal involvement [5].

The typical upper respiratory symptoms are nasal stuffiness, sinus pain, nasal discharge, ulcerations, and epistaxis. Cough, hemoptysis, shortness of breath, and pleuritic chest pain are common lower respiratory tract symptoms. Hematuria is seen with renal involvement. Eye pain is a common symptom in about 50% of cases of GPA from scleritis and conjunctivitis [6].

Patients may also have non-specific symptoms like generalized fatigue, fever, malaise, weight loss, joint pains, and muscle pains. The examination findings differ based on the extent and severity of organ involvement and the severity of the disease.

Few cases of isolated pulmonary GPA are published in medical literature, and other lung pathologies like tuberculosis, sarcoidosis, bacterial, fungal infections, and malignancies must be ruled out before diagnosing limited GPA [7]. We had ruled them out in our case. GPA can even present with severe forms like acute glomerulonephritis, pulmonary hemorrhage, cerebral vasculitis, peripheral and cranial neuropathy, pericarditis, myocarditis, and gastrointestinal bleeding.

The exact cause of GPA is not very well understood. ANCA appears to be the primary inflammatory antibody in GPA. It reacts with proteinase 3 (an enzyme prevalent in neutrophil cytoplasm) and activates neutrophils. The sensitivity of ANCA is 66%, and specificity is 98% in GPA. Rarely GPA may be ANCA negative. Genetic defects in alpha-1 antitrypsin, proteinase 3 gene, major history compatible complex class II gene, bacterial infections (staphylococci), virus (hepatitis C, cytomegalovirus, and Epstein-Barr virus), and medications like hydralazine, allopurinol, and phenytoin are other risk factors for GPA [8].

Histologically GPA starts with the formation of neutrophilic microabscesses. These granulomas are not well-formed like tuberculosis. They consist of giant cells surrounded by lymphocytes, plasma cells, and dendritic cells. They damage the surrounding tissues, mucosa, bone, and cartilage resulting in necrosis and the permanent damage of adjacent tissue [9]. In the lung, these inflammatory cells cause liquefaction necrosis and can resemble an abscess. Kidney biopsy shows focal necrotizing glomerulonephritis with cellular crescents, glomerular thrombi, and rarely granulomas [10].

In our case, the predominant symptom is cough and shortness of breath with some fatigue and joint stiffness. Systemic examination was normal except for lung examination and abnormal lung imaging. After ruling out infectious and malignant etiologies on at least two occasions based on repeated cultures and cytology examination, the development of recurrent sterile lung abscess made us think this is probably an inflammatory condition mimicking infection. So a formal decision was made to surgically explore and get tissue diagnosis and immunofluorescence antibody assay to establish a definitive diagnosis. After this, we have come to the conclusion that the patient had a limited GPA in the absence of upper respiratory tract and renal involvement.

In patients with suspected GPA, evaluation should include a detailed history, examination, laboratory, imaging, and histopathologic workup. The evaluation should include a complete assessment of different organ systems and the extent of each organ involved. Basic laboratory workup including complete blood count, serum electrolytes, kidney and liver function test, and urinalysis. A tissue diagnosis is needed for provisional diagnosis; the lung is the most common site for biopsy, followed by the kidney. An immunofluorescence antibody assay (serologic workup) should be done to confirm the diagnosis of GPA. The presence of granuloma and association with proteinase 3 ANCA favors GPA over microscopic polyangiitis where there is no specific granuloma formation and is associated with myeloperoxidase ANCA [11].

The 2022 American College of Rheumatology (ACR)/European Alliance of Associations for Rheumatology (EAAR) classification criteria include three clinical and seven laboratory, imaging, and biopsy criteria. Each criterion has specific scores, as shown in Table 1 [12].

**Table 1:** 2022 American College of Rheumatology/European Alliance of Associations for Rheumatology classification criteria for granulomatosis with polyangiitis. A score of >5 is needed to classify granulomatosis with polyangiitis. C-ANCA: antineutrophil cytoplasmic autoantibody, cytoplasmic; p-ANCA: perinuclear anti-neutrophil cytoplasmic antibodies

Criteria	Score
Nasal involvement - bloody discharge, ulcers, crusting, blockage, congestion, septal defect, or perforation	+3
Cartilaginous involvement (inflammation of the ear/nose/throat), hoarse voice, stridor, saddle nose deformity, and endobronchial involvement	+2
Conductive or sensorineural hearing loss	+1
Laboratory, imaging, and biopsy criteria	
Positive test for C-ANCA, or antiproteinase 3 antibodies	+5
Pulmonary nodules, mass, or cavitation on CT imaging	+2
Granuloma, extravascular granulomatous inflammation, or giant cells on biopsy	+2
Inflammation, consolidation, or effusion of the nasal/paranasal sinuses or mastoiditis on imaging	+1
Posse-immune glomerulonephritis on biopsy	+1
Positive test for p-ANCA or anti-myeloperoxidase antibodies	-1
Blood eosinophil count more than 1x10^9^/L	-4

Our patient had no clinical criteria but had three of seven laboratory criteria. Five points for C-ANCA, two points for lung nodules, and two points for granuloma on histologic examination, thus confirming GPA.

Treatment for GPA is based on the severity of the disease. The limited form of GPA has an excellent prognosis. Treatment of GPA is given in two phases, the induction and maintenance phases. Historically, cyclophosphamide, combined with pulse dose steroids, is used for induction in severe forms of the disease. Rituximab, an anti-CD20 B-cell depleting monoclonal antibody, is non-inferior compared to cyclophosphamide for induction but may even be superior in relapse and is the current treatment of choice [13]. In patients with non-severe disease, methotrexate and steroids are commonly used for induction. In fulminant disease with rapidly declining kidney function, pulmonary hemorrhage complicating respiratory compromise, plasmapheresis is indicated [14]. Maintenance therapy is initiated three to six months after remission is achieved. Methotrexate, rituximab, and azathioprine are used as maintenance drugs; azathioprine has the best safety profile, and steroids are slowly weaned off [15].

Because of limited and mild disease, our patient was started on methotrexate, and subsequent follow-ups demonstrated improvement in symptoms. A follow-up chest x-ray did not show any new lesions. At the time of writing this report, the patient had been on maintenance of methotrexate for ten months, and she did not show any signs of bone marrow suppression or hepatotoxicity. Long-term immunosuppression medications might contribute to adverse events and should be monitored.

GPA is associated with significant morbidity and mortality because of the disease, delay in diagnosis, and immunosuppressive medications. Patients with limited pulmonary disease, however, have a good prognosis. Multiorgan involvement, specifically severe renal disease, has an inferior prognosis and a high mortality rate [16].

Complications from the disease include hearing loss, vision loss, saddle nose deformity, hypoxic respiratory failure, and kidney injury resulting in end-stage renal disease. Toxic effects of immunosuppressive treatment include infections, infusion reactions, and myelodysplastic syndromes.

## Conclusions

The limited form of granulomatosis with polyangiitis is a rare autoimmune vasculitis predominantly involving the lower respiratory tract. Though commonly seen in Caucasians, it should be suspected in people with recurrent culture-negative empyema and lung mass with no other risk factors in all ethnic groups. Diagnosis is confirmed with tissue biopsy and immunofluorescence antibody assay. A high index of suspicion, early confirmation, and prompt initiation of treatment with immunosuppression will improve patient outcomes.
